# Nebulisation of Paclitaxel, Sotatercept and Iloprost for pulmonary hypertension for lung cancer. From *In vitro* to *In vivo*

**DOI:** 10.7150/jca.90732

**Published:** 2024-01-01

**Authors:** Paul Zarogoulidis, Dimitris Petridis, Haidong Huang, Chong Bai, Georgia Pitsiou, Dimitris Matthaios, Eleni-Isidora Perdikouri, Vasilis Papadopoulos, Savvas Petanidis, Christoforos Kosmidis, Wolfgang Hohenforst-schmidt, Konstantinos Porpodis, Nikos Kougas, Panagoula Oikonomou, Christina Nikolaou, Charalampos Charalampidis, Chrysanthi Sardeli

**Affiliations:** 1 Pulmonary Department, General Clinic Euromedica, Thessaloniki, Greece.; 2 3rd University Surgery Department, ``AHEPA`` University Hospital, Thessaloniki, Greece.; 3 Department of Food Technology, School of Food Technology and Nutrition, Alexander Technological Educational Institute, Thessaloniki, Greece.; 4 Department of Respiratory and Critical Care Medicine, Changhai Hospital, Navy Military Medical University, Shanghai, 200433, China.; 5 Pulmonary Department, ``G. Papanikolaou`` General Hospital, Aristotle University of Thessaloniki, Greece.; 6 Oncology Department, General Hospital of Rhodes, Greece.; 7 Oncology Department, General Hospital of Volos, Greece.; 8 Oncology Department, University General Hospital of Larissa, Greece.; 9 Department of Medicine, Laboratory of Medical Biology and Genetics, Aristotle University of Thessaloniki, Thessaloniki, Greece.; 10 Sana Clinic Group Franken, Department of Cardiology / Pulmonology / Intensive Care / Nephrology, ''Hof'' Clinics, University of Erlangen, Hof, Germany.; 11 Rheumatology Department, Ippokrateio University General Hospital, Thessaloniki, Greece.; 12 Surgery Department, Democritus University of Thrace, Alexandroupolis, Greece.; 13 Department of Pathology, University of Cyprus, Cyprus.; 14 Department of Pharmacology & Clinical Pharmacology, School of Medicine, Aristotle University of Thessaloniki, Thessaloniki, Greece.

**Keywords:** Paclitaxel, iloprost, sotatercept, jet-nebulizers, ultrasound nebulizers, pulmonary hypertension

## Abstract

**Background:** Pulmonary hypertension is common symptom among several diseases. The consequences are severe for several organs. Pulmonary hypertension is usually under-diagnosed and the main symptom observed is dyspnea with or without exercise. Currently we have several treatment modalities administered orally, via inhalation, intravenously and subcutaneously. In advanced disease then heart or lung transplantation is considered. The objective of the study was to investigate the optimum method of aerosol production for the drugs: iloprost, paclitaxel and the novel sotatercept.

**Materials and Methods:** In our experiment we used the drugs iloprost, paclitaxel and the novel sotatercept, in an experimental concept of nebulization. We performed nebulization experiments with 3 jet nebulizers and 3 ultrasound nebulizers with different combinations of residual cup designs, and residual cup loadings in order to identify which combination produces droplets of less than 5μm in mass median aerodynamic diameter.

**Results:** We concluded that paclitaxel cannot produce small droplets and is also still very greasy and possible dangerous for alveoli. However; iloprost vs sotatercept had smaller droplet size formation at both inhaled technologies (1.37<2.23 and 1.92<3.11, jet and ultrasound respectively). Moreover; residual cup designs C and G create the smallest droplet size in both iloprost and sotatercept. There was no difference for the droplet formation between the facemask and cone mouthpieces.

**Discussion:** Iloprost and sotatercept can be administered as aerosol in any type of nebulisation system and they are both efficient with the residual cups loaded with small doses of the drug (2.08 and 2.12 accordingly).

## Introduction

In pulmonary hypertension (PAH) increased pulmonary vascular resistance (PVR) is observed, which usually leads to right ventricular heart failure. The main symptoms from increased PVR are: dyspnea, fatigue, orthopnea, dizziness, fainting, non-productive cough, peripheral edema angina pectoris, finally in severe cases leg swelling.^1^ Pulmonary hypertension progressive and is considered a fatal condition in the final stages. Decreased exercise tolerance and heart failure is also observed. Symptoms usually develop over the years and therefore diagnosis is delayed. However; there are patients which develop at early stages hemoptysis or have fainting symptoms and/or even syncope. In the case of *venous* hypertension shortness of breath is observed while lying flat, while in the case of pulmonary *arterial* hypertension this symptom is not observed. There are 5 types of PAH and therefore several tests have to be performed to distinguish pulmonary *arterial* hypertension from *venous* hypertension. Tests include blood tests to exclude HIV, pulmonary function tests, autoimmune diseases, arterial blood gas measurements, electrocardiography, liver disease, ventilation-perfusion or V/Q scanning to exclude chronic thromboembolic pulmonary hypertension and CT-angiography of the thorax. Lung biopsy is performed only in the case of an underlying interstitial lung disease. An easy method to evaluate the clinical improvement of these patients is the six-minute walk test (6MWT). It has been previously observed that improvement of the 6MWT values correlates with increased survival benefit. Furthermore; Blood BNP levels are considered marker for disease stability or progression for these patients.^2^ PAH can be evaluated estimated in the everyday clinical practice with ultrasound echocardiography, however; the gold standard is the pressure measurement with a Swan-Ganz catheter. Right-sided cardiac catheterization is required for the diagnosis of pulmonary arterial hypertension.^3^ Normal pulmonary arterial values are set to be between 8-20 mm Hg (1066-2666 Pa) at rest. Pulmonary hypertension diagnosed when the mean pulmonary artery pressure exceeds 25 mm Hg at rest. In order to administer proper treatment we have to evaluate whether the PAH is arterial, venous, hypoxic, miscellaneous or thromboembolic. For patients with left heart failure or hypoxemic lung diseases (groups II or III pulmonary hypertension), we should not administer endothelin antagonists, phosphodiesterase inhibitors, or prostanoids.^4^ The first line treatment for pulmonary arterial hypertension is considered with; digoxin, diuretics, oxygen therapy and oral anticoagulants. Moreover; high dose calcium channel blockers can be administered for idiopathic pulmonary arterial hypertension patients.^5^, ^6^ There are several novel drugs investigated for PAH and the main method of effectiveness evaluation is still the 6MWT.^7^ Tyrosine kinase inhibitors have been evaluated as a treatment for pulmonary hypertension.^8^-^10^ Imatinib has been recently investigated against pulmonary hypertension.^11^-^15^ Paclitaxel has been recently evaluated as a remodeling agent for PAH.^16^ Sotatercept has been recently been evaluated in a clinical trial with favorable results (the PULSAR study)^17^. We investigated whether the drugs paclitaxel, sotetercept and iloprost could be administered as aerosol with jet and ultrasound nebulizers. Moreover; we evaluated the optimal combination of residual cup design, residual cup loading and nebulizer, in order to produce droplets of ≤5μm.

## Materials and methods

### Drugs

The drugs Ventavis® (iloprost, 15mg/ml, Bayer), Paxene® (paclitaxel, 150mg/25ml, Norton Healthcare, Ltd) and Sotatercept® (sotatercept 0.3mg/ml Merck & Co., Inc).

### Aerosol Production Systems

#### Jet-Nebulizers and residual cups

**Figure 1:**
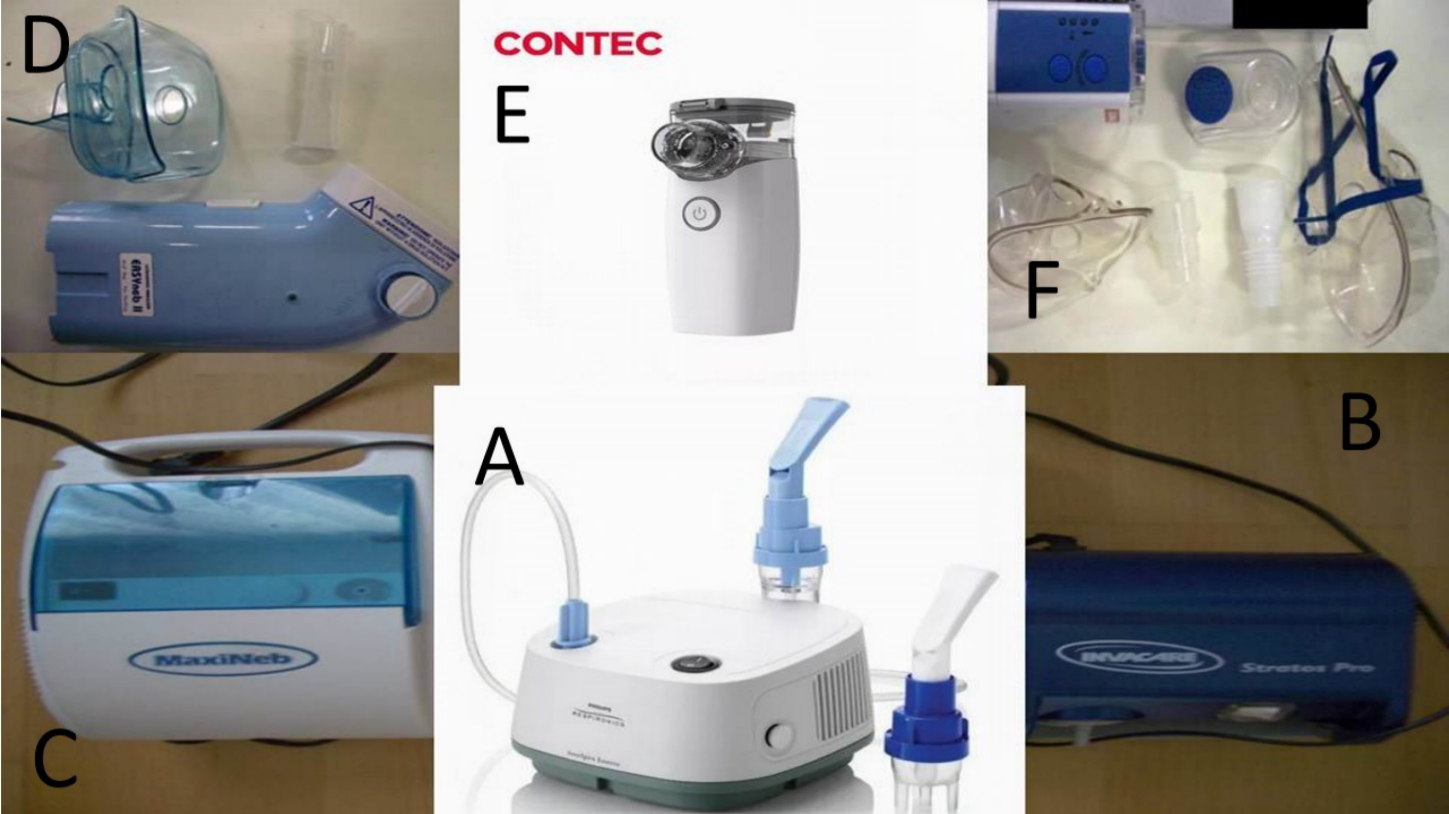
A) Philips jet-nebuliser, B) Invacare jet-nebuliser, C) Maxineb jet-nebuliser, D) Easybeb ultrasound nebuliser, E) Contec ultrasound nebuliser, F) Omron ultrasound nebulizer.

Three jet-nebulizers were chosen for the experiment: a) A Philips Respironics Innospire Essence Compressor Nebulizer System - SideStream Technology, Compressor max. pressure 317 kPa (46 psi), Respironics New Jersey, Inc, Parsippany, NJ 07054 USA, b) Maxineb^®^ (6 L/min and 35 psi), Hof, Germany and c) Invacare® (4-8 liters-minute and 36 psi) (Figure 1). We used 7 different residual cups. Four had a capacity of no more than 6 ml and two with a capacity no more than 10 ml.

**Figure 2:**
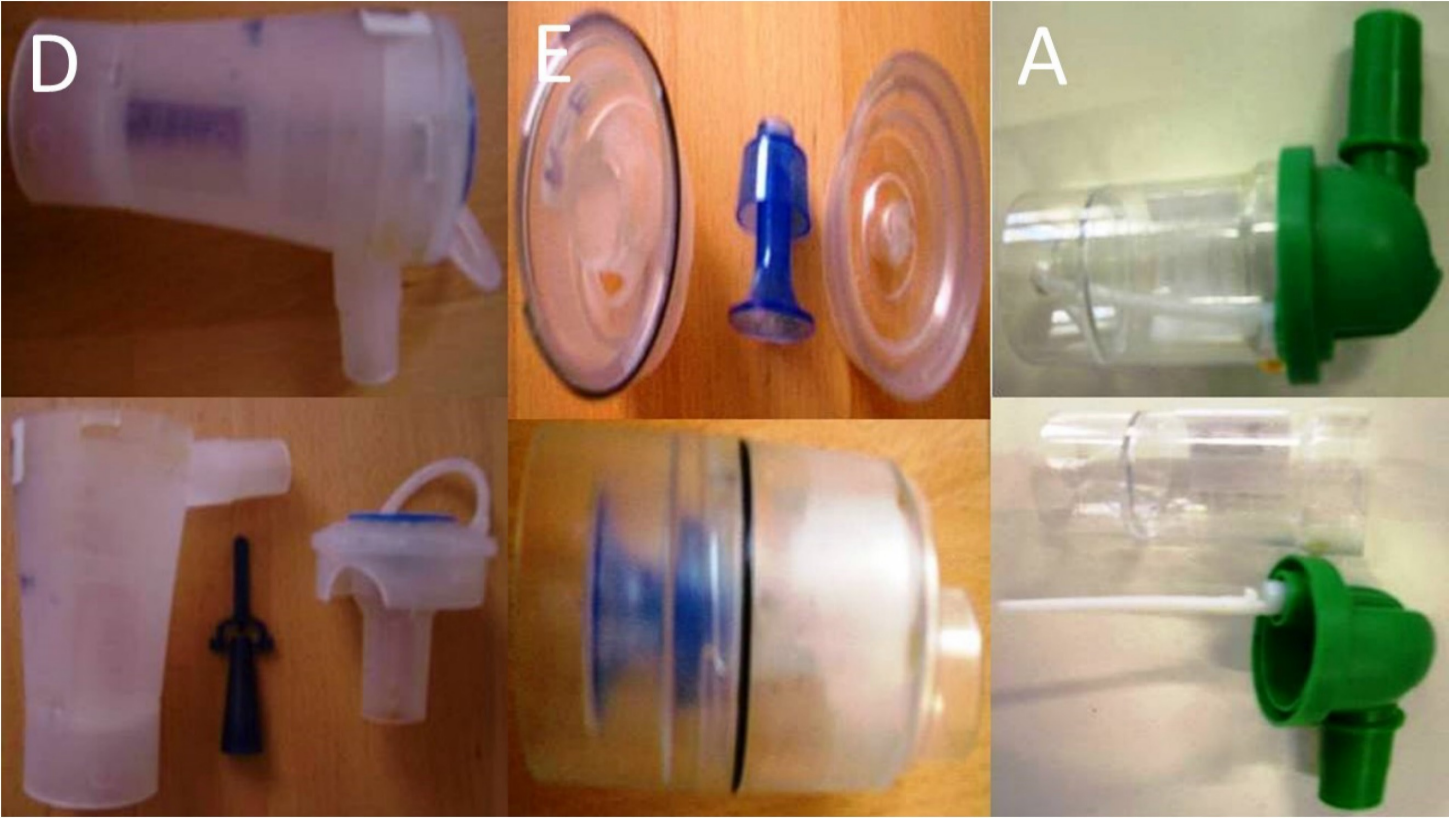
Cup D 9cm in length and 4.5cm in diameter, Cup E 9cm in length and 4.5cm in diameter and Cup A 9cm in length and 4.5cm in diameter Large residual cups.

**Figure 3:**
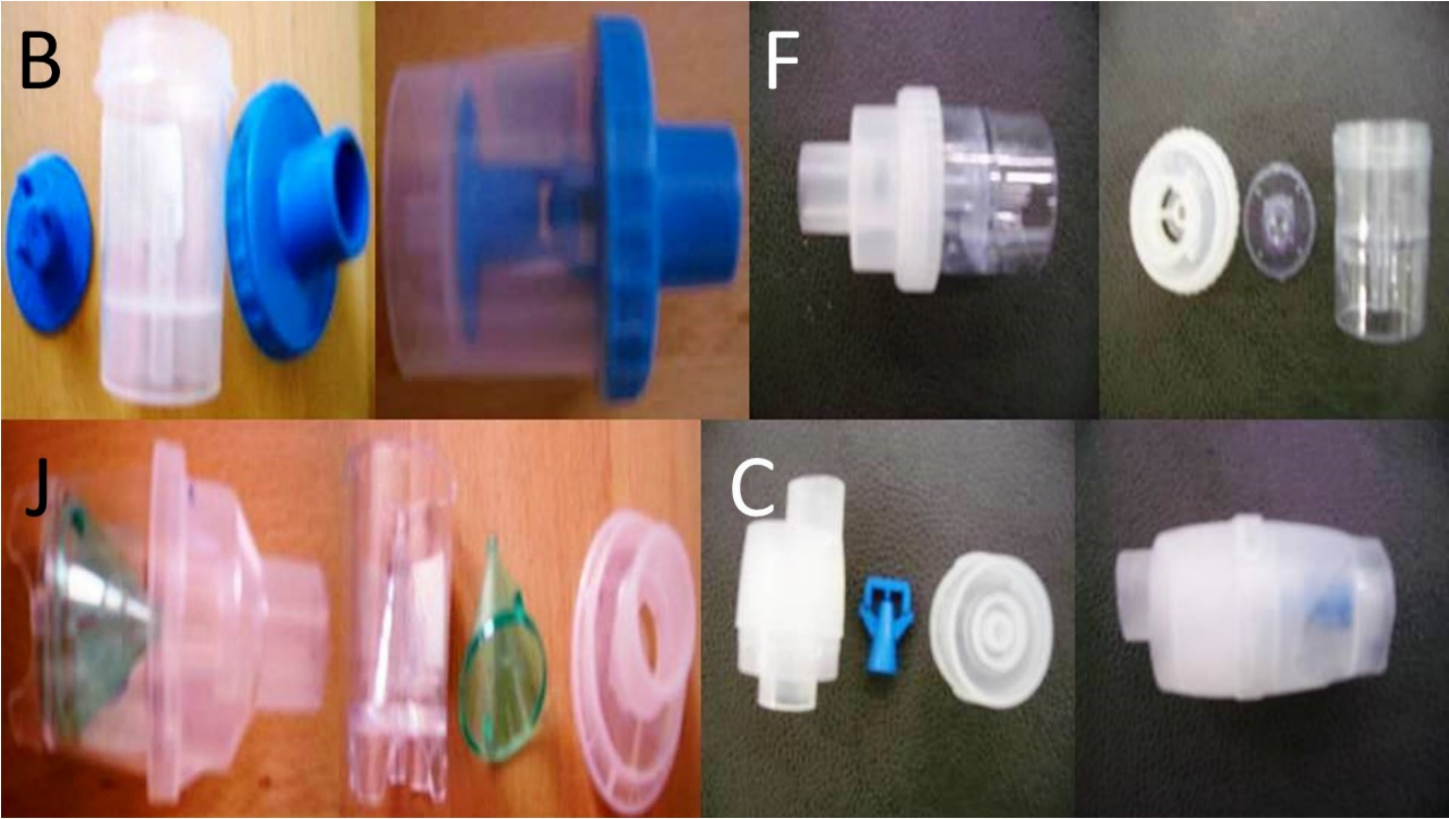
Cup B 5cm in length and 3cm in diameter, Cup F 5cm in length and 3cm in diameter and Cup J 5cm in length and 3cm in diameter Small and Cup C 5cm in length and 3cm in diameter residual cups.

For large cups we used the letters; A, D and E. For small residual cups we used the letters; C, F, B and G. (Figure 2,3) Large residual cups were used with a capacity of 2-8 mls. The residual cup loadings were 2,4,6 and 8ml (8ml only for large cups).

### Ultrasound Nebulizers

**Figure 4:**
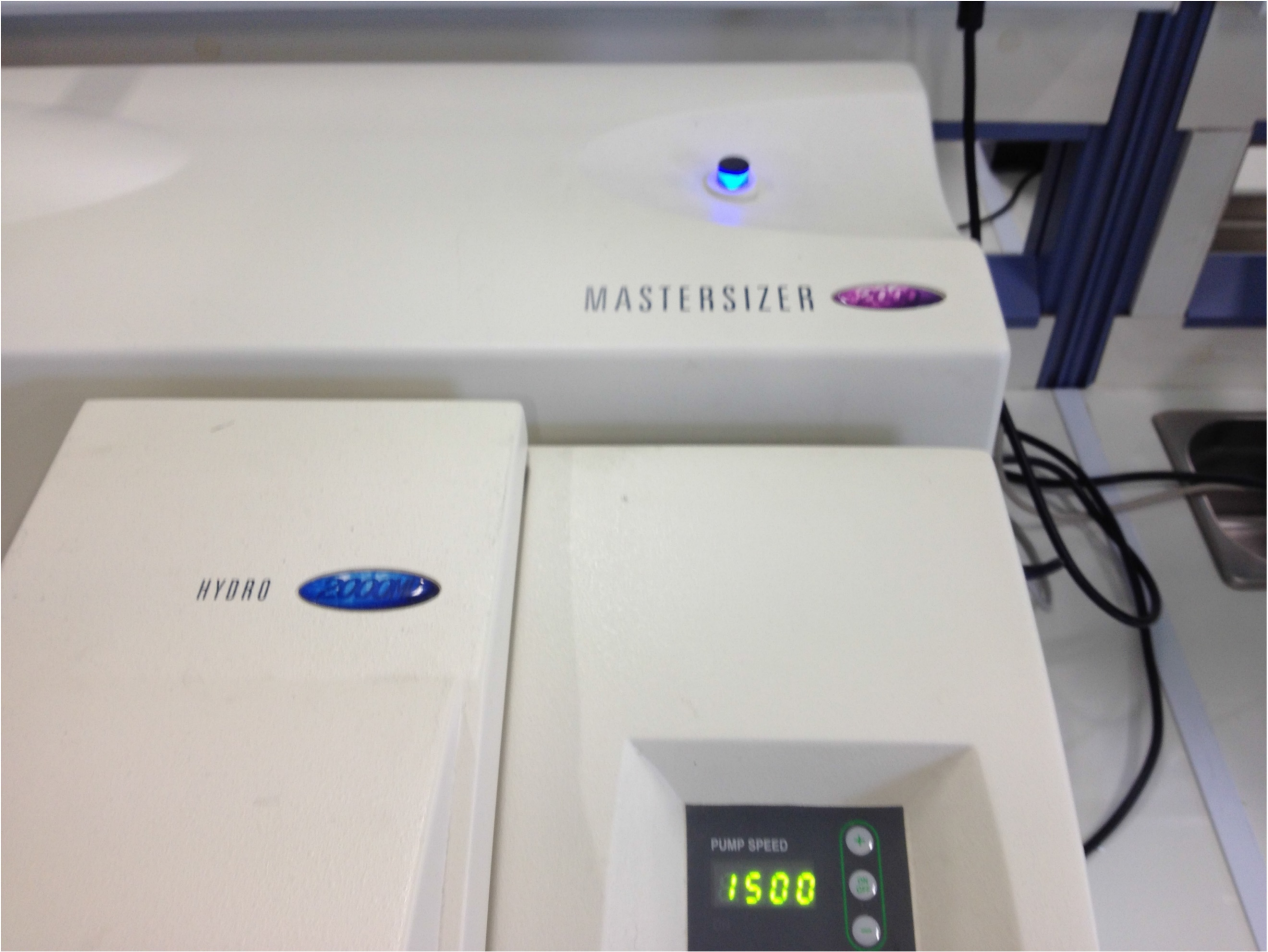
Matersizer 2000.

Three ultrasound nebulizers were purchased for our experiment. An Omron® NE-U07, Tokyo, Japan. Compact and weighs less than 350gm, includes a 10ml medication cup. Generates uniform micromillimetre-sized vapor particles. A Contec NE-M01 portable handheld Mesh Nebulizer, CONTEC medical systems Co., Ltd., UK was also purchased. The third ultrasound nebulizer was a portable EASYneb® II, FLAEMNUOVA, Martino, Italy. The loadings were 2 and 4 mls based on the residual cup capacity of each of the three ultrasound nebulizers (Figure 4).

### Droplet Measurement

We used a Malvern Mastersizer 2000 apparatus (Malvern Instruments Ltd, Malvern, Worcestershire, UK) equipped with a Scirocco module (Malvern Instruments Ltd, Malvern, Worcestershire, UK) in order to calculate the size distribution of the droplets and their mean diameter (*d*_32_). A refractive index of 1.33 was been used for the sprayed droplets. Three experiments were performed for each combination.^18^-^22^

### Statistical analysis

#### Jet technology

There were 4 factors affecting the droplet size: two drugs (iloprost and sotatercept), 3 nebulizers (INVACARE, RESPIRONICS, MAXINEB), 7 residual cup/residual cup designs (A to G) and 3 loadings (2, 4, 6ml). A four factor ANOVA was performed with 0.05 probability reference level. Pair-wise statistically significant differences between means were examined using the 95% confidence intervals. Again, an ANOVA test was performed for the residual cups (A, D, E) that were filled with 8ml dose using the same drugs and nebulizers.

#### Ultrasound technology

Iloprost and sotatercept along with the nebulizers (EASYNEB, CONTEC, OMRON) were investigated at 2 dose levels (2, 4ml) for their impact on particle size formation.

### Mice

One hundred twenty BALBC mice age 9 weeks old were purchased from the experimental laboratory of ``Theiageneio`` Anticancer Hospital, and were divided in 3 groups. The Institute has the following authorization for production and experimentation of mice EL 25 BIO 011 and EL 25 BIO 013. The mice included were isolated (1 per cage) in a temperature-controlled room on 12-hour light-dark cycle and were allowed free access to food and water. The Lewis lung carcinoma cell line was obtained by ATCC (CRL-1642™). The cells were routinely cultured in 25-cm2 tissue culture flasks containing RPMI (ATCC, 30-2002) supplemented with 10% fetal bovine serum (Biochrom) according to the supplier's instruction. The cell line was incubated at 37^ο^ C in 5% CO2. The doubling time of the cell line was 21 hours.^33^ At confluence, cells were harvested with 0.25% trypsin and then were re-suspended at 1,5×10^6^ cells in 0.15 ml PBS (Phosphate Buffered Saline, Dulbecco, Biochrom) which was injected in mice. The back was inoculated subcutaneously (27-guage needle, 1,5×10^6^ cells). The tumor volume was measured once weekly using bidimensional diameters (caliper) with the equation V=1/2*ab*^2^, where the *a* represents the length and *b* the width (mm^3^). The tumor was grown on the back of the mice. The animals were randomly divided into six groups of 20, when the tumor volume reached ~100mm^3^. The mice were divided into six groups as follows: a) paclitaxel, b) sotatercept and c) iloprost.

### Aerosol administration

**Figure 5:**
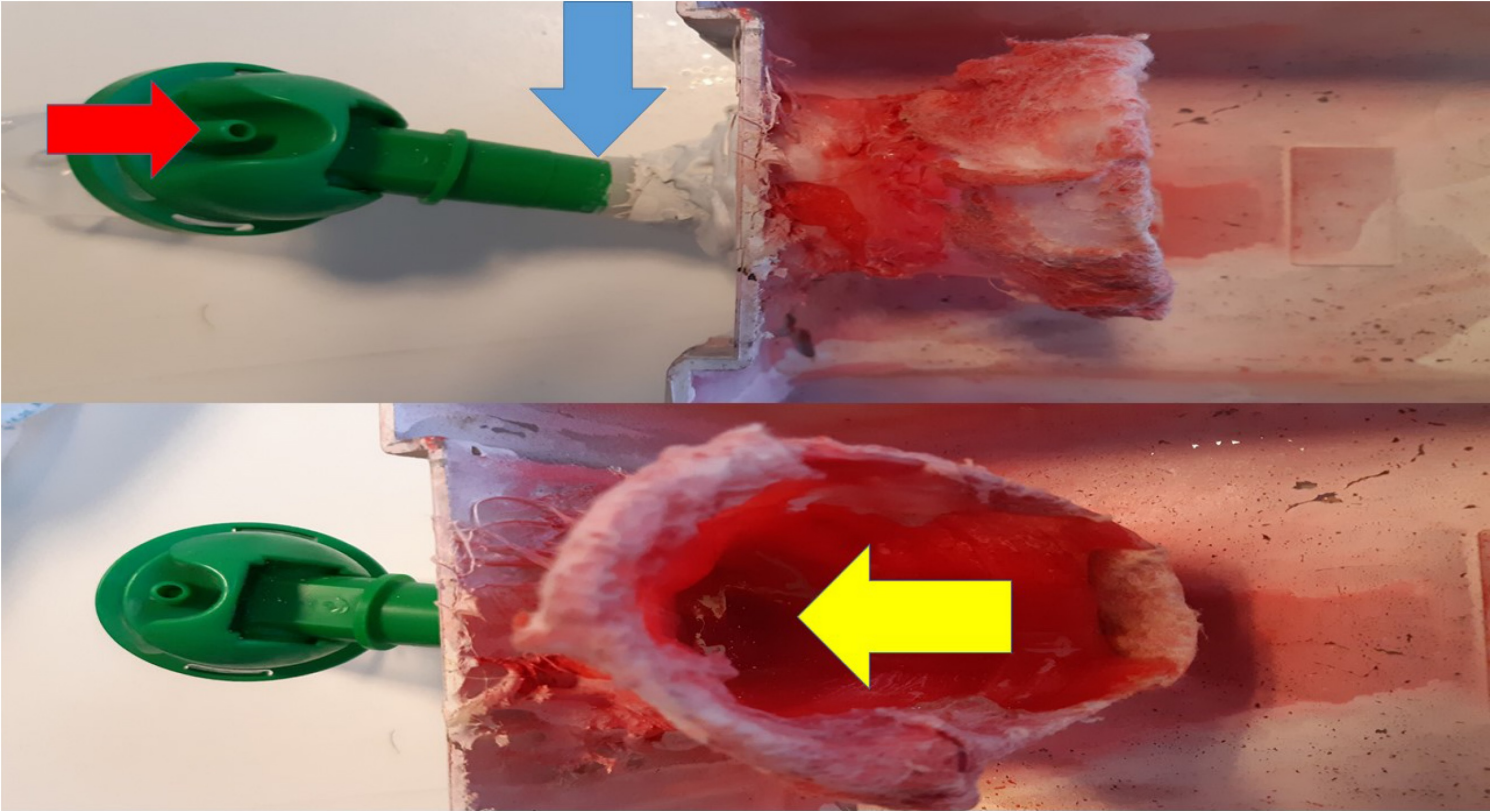
Red arrow indicates the connection tip for nebulizer, the blue arrow the connection between produced aerosol and inlet, Yellow arrow indicates the inlet part where the mice introduce their nose.

Aerosol was administered with the cage presented in figure 5. This cage was specifically designed for this study with a special inlet that connects with the nebulizer reservoir. For every drug group, after initial experiments which are mentioned in detail, we chose the optimum combination that creates the smallest droplet. This information is provided in the results section below.

### Nebuliser Results

#### Jet technology

The main factors affecting the droplet formation were the drugs and the residual cup designs (Table 1, p-values <0.001). Iloprost reduced the mean droplet size down to 1.37 μm when compared to sotatercept (2.23).

The residual cups C and G have the lowest droplet size production 1.32 and 1.37 respectively.

The rest of the residual cups have equal mean size droplets produced, however; with higher mean droplets. In the 6ml loading capacity the mean droplet size was higher than ≤6mls loading (p=0.048).

Sotatercept produced larger mean droplets (2.57) (p=0.039) when combined with the nebulizer MAXINEB. Regarding loading with 8mls, it was observed that the mean droplet size did not differ versus 6mls and the experimentation was abandoned from further investigation.

#### Ultrasound technology

The drug, residual cup loading and mouthpiece, did not exert any statistically significant effect on particle size (Table 2, p-values, 0.020, 0.036 and 0.043 accordingly). Iloprost again produces smaller mean droplet size than sotatercept (1.92<3.11, Table 3). The facemask produced slightly lower mean droplet size than the cone inlet (2.12<2.91). Moreover; the 2ml dose versus the 4ml (2.08<2.95). The cone inlet produced small mean droplets compared to the facemask with a droplet size between (2.10 and 2.05) (cone inlet/facemask) (see also Table 2, p=0.038).

Iloprost produces smaller mean droplet size with both jet and ultrasound nebulisers (1.37<2.23 and 1.92<3.11, jet and ultrasound respectively) and, even smaller mean droplet size with jet devices (1.37<1.92). Residual cup designs C and G contribute in the most efficient way in the production of the small mean droplet size uniquely and equally for both drugs. It was observed that sotatercept produced with the residual cup design C (1.37 instead of 2.23). Moreover; 2ml loading produces the smallest mean droplet size both for the facemask and cone inlet. The facemask and low 2ml residual cup loading is the best combination choice (2.08 and 2.12 accordingly). We could not assess the results of paclitaxel since paclitaxel formed some kind of colloid substance and the mastersizer operation was blocked and we had to clean the lances again and again in order for the equipment to work again. The colloid substance that was formed was due to the drug properties.

### Mice results

**Figure 6:**
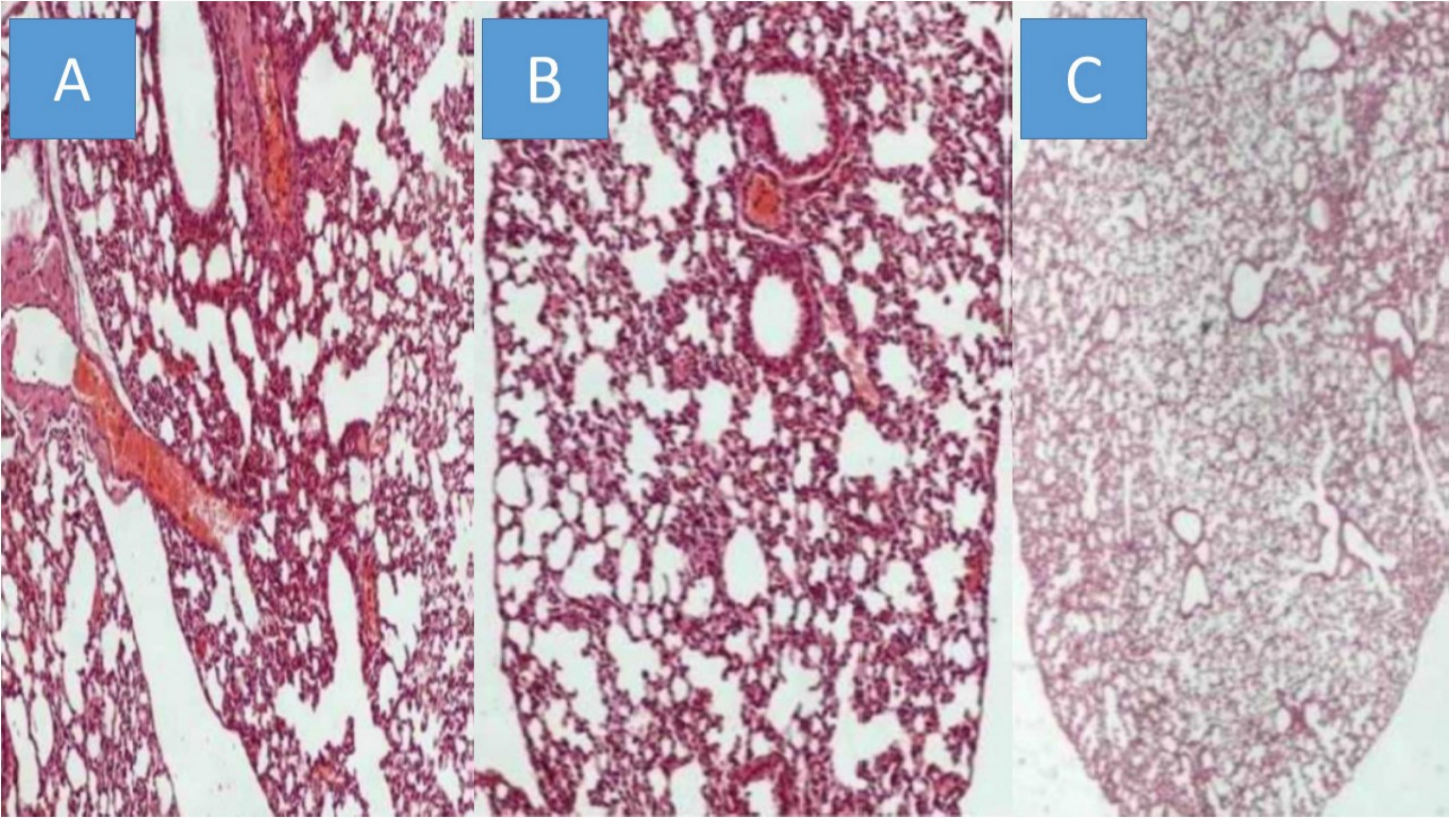
** A)** Paclitaxel group, **B)** Sotatercept group, and **C)** Iloprost group all x 200 magnification.

All animals were killed on the 20^th^ day after the initiation of the administration. The mean volume values were recorded throughout the experiment and upon death or the last day of death the final measurement was included in our data for mean tumor volume measurement extraction. The mean volume measurements are as follow for each group (mm^3^): a) 2132.4, b) 2361.2, c) 346.32. We used the following technique to measure the drug concentrations in the lung tissue of the mice. In the first step, the sample mass to be digested can be up to ca. 1.000 g however in some of the samples lower mass (e.g. 0.1500 g) was available and was finally digested. The procedure involved weighing of the samples in Teflon® (DuPont, DE, USA) crucibles, addition of 6 mL concentrated HNO_3_, and heating in a steel autoclave (Berghof, BTR 941, Eningen, Germany, six-position aluminum block). High pressure conditions in the closed vessels assist the decomposition of the sample tissues which is completed in less than 2 h at 130 ^0^C. The obtained solution was diluted to 25 mL by double de-ionized water. The analytical instrument that was used was an ICP-AES Optima 3100 XL (Perkin-Elmer, MA, USA) operated in axial-viewing mode and equipped with a segmented array charge-coupled device (SCD) as detector. The drugs were detected in all mice in their lungs, and therefore we had proof of concept. However; as it was anticipated emphysema damage was observed mostly in the paclitaxel group, less in sotetercpt group and even less in iloprost group. Figure 6. In any case the drugs administered, their consistency has not been created for lung administration as aerosol.

## Discussion

Our respiratory system has different defense mechanisms like; a thick layer of the mucus, the beating cilia and finally macrophages which interact with the aerosol droplet deposition.^23^ Therefore an aerosolized drug has to efficiently pass firstly those factors. Furthermore; the aerosol droplets have to be of a mass median aerodynamic diameter ≤5μm. Due to the increased humidity of the respiratory system >90% the aerosol droplets tend to increase in size by almost 50% during their passage to the lower respiratory tracts.^23^ Inhaled drug administration has been observed to be equally effective for many diseases due to the local effect with less drug dosage and therefore less adverse effects, such as in the case of chronic obstructive pulmonary disease (COPD). Inhaled insulin is another example. 24-29 Inhaled antibiotics and inhaled inhibitors for pulmonary hypertension are on the market 22, 30. There are still safety concerns for the lung parenchyma that are being investigated as in the case of tyrosine kinase inhibitors.^31^, ^32^ Tyrosine kinase inhibitors have been used for a decade to target non-small cell lung cancer with epidermal growth factor receptors. 33 Recently it was presented that TKIs are potent acute pulmonary vasodilators.^34^ Sorafenib a multikinase inhibitor has been investigated as aerosol against vascular remodeling from arterial hypertension.^35^,^36^ Imatinib another TKI inhibitor was successfully administered against pulmonary hypertension in a patient with chronic eosinophilic leukemia.^37^ In another study imatinib was used directly against arterial hypertension.[14, 38, 39]A major drawback with inhaled drugs was observed when respiratory tract infection occurred and the pharmacokinetics of the drugs changed.^24^ The factors that mostly influence the aerosol droplet production are; a) jet-nebuliser flow rate^40^, b) viscosity^40^, c) tapping of the residual cup during nebulisation^41^, ^42^, d) chemical formula^43^, ^44^, e) residual cup loading^45^, f) residual cup filling upon initiation of nebulisation^41^, g) design of the residual cup^46^, h) charge of the drug molecules^47^, i) concentration of drug solution and j) surface tension. Moreover; the salt concentration within the chemical structure of the drug formulation is responsible for the absorption of water from the environment. Platelet derived growth factor (PDGF) inhibitors have been also used against PAH.^48^ Vascular endothelial growth factor inhibitors have been successfully used against PAH^49^, ^50^ It has been proposed that inhibiting the PDGF pathway is more efficient against PH since fibrinogenesis is blocked simultaneously^51^ The rho-kinase (ROCK) inhibitors 52, 53 and dasatinib has been used to induce PAH.^54^ A new Syk kinase inhibitor is in being developed for inhalation by Pfizer and is being investigated in a Phase I study.^55^ Rapamycin has been proposed as an antiproliferative agent for smooth muscle cells implicating that it can be used against PAH.^56^ In our study iloprost produces smaller droplets compared to sotatercept, however; both drugs can be administered as aerosol (1.37<2.23 and 1.92<3.11, jet and ultrasound respectively). Jet devices produce smaller droplets for both drugs (1.37<1.92). Cup designs C and G produce the smallest drug aerosol droplets for both drugs. The residual cup C design has the ability to produce small aerosol droplet size for sotatercept when compared with other residual cup designs (1.37 instead of 2.23). Finally, at 2ml residual cup filling the facemask and cone mouthpieces perform equally at their best (2.08 and 2.12 accordingly). We concluded that paclitaxel cannot produce small droplets and is also still very greasy and possible dangerous for alveoli.

## Figures and Tables

**Table 1:** Analysis of variance of four fixed factors and their interactions on MMAD. Statistically significant effects are shown in bold.

Effect	SS	DF	MS	F	p
DRUG	**23,50**	**1**	**23,50**	**69,19**	**0,000**
NEBULIZER	1,19	2	0,60	1,75	0,194
RESIDUAL CUP	**13,01**	**6**	**2,17**	**6,39**	**0,000**
LOADING	**2,35**	**2**	**1,17**	**3,46**	**0,048**
DRUG*NEBULIZER	**2,52**	**2**	**1,26**	**3,71**	**0,039**
DRUG*RESIDUAL CUP	**14,12**	**6**	**2,35**	**6,93**	**0,000**
NEBULIZER*RESIDUAL CUP	7,00	12	0,58	1,72	0,126
DRUG*LOADING	0,28	2	0,14	0,41	0,668
NEBULIZER*LOADING	1,58	4	0,39	1,16	0,352
RESIDUAL CUP*LOADING	3,61	12	0,30	0,89	0,571
DRUG*NEBULIZER*RESIDUAL CUP	**9,45**	**12**	**0,79**	**2,32**	**0,038**
DRUG*NEBULIZER*LOADING	1,79	4	0,45	1,32	0,293
DRUG*RESIDUAL CUP*LOADING	3,36	12	0,28	0,83	0,625
NEBULIZER*RESIDUAL CUP*LOADING	6,69	24	0,28	0,82	0,684
Error	8,15	24	0,34		

**Table 2:** Analysis of variance of four fixed factors and their interactions on MMAD. Statistically significant effects are shown in bold.

Effect	SS	DF	MS	F	p
DRUG	**8,58**	**1**	**8,58**	**49,01**	**0,020**
NEBULIZER	3,12	2	1,56	8,93	0,101
LOADING	**4,60**	**1**	**4,60**	**26,29**	**0,036**
MOUTHPIECE	**3,79**	**1**	**3,79**	**21,65**	**0,043**
DRUG*NEBULIZER	3,20	2	1,60	9,13	0,099
DRUG*LOADING	1,17	1	1,17	6,71	0,122
NEBULIZER*LOADING	1,79	2	0,90	5,12	0,163
DRUG*MOUTHPIECE	0,50	1	0,50	2,85	0,233
NEBULIZER*MOUTHPIECE	0,85	2	0,43	2,44	0,291
LOADING*MOUTHPIECE	**4,27**	**1**	**4,27**	**24,43**	**0,039**
DRUG*NEBULIZER*LOADING	0,25	2	0,13	0,73	0,579
DRUG*NEBULIZER*MOUTHPIECE	3,22	2	1,61	9,21	0,098
DRUG*LOADING*MOUTHPIECE	0,30	1	0,30	1,70	0,322
NEBULIZER*LOADING*MOUTHPIECE	0,49	2	0,25	1,41	0,416
Error	0,35	2	0,18		

**Table 3:** Mean values of MMAD and 95% confidence intervals for drugs, mouthpiece devices, loading levels and nebulizers calculated from the ANOVA's error mean square.

DRUG	Mean	-95%	95%	N
SOTATERCEPT	3,11	2,59	3,63	12
ILOPROST	1,92	1,40	2,44	12
MOUTHPIECE	Mean	-95%	95%	N
FACEMASK	2,12	1,60	2,64	12
CONE INLET	2,91	2,39	3,43	12
LOADING	Mean	-95%	95%	N
2	2,08	1,56	2,60	12
4	2,95	2,43	3,47	12
NEBULIZER	Mean	-95%	95%	N
EASYNEB	2,88	2,25	3,52	8
CONVEC	2,64	2,00	3,27	8
OMRON	2,02	1,39	2,66	8
